# Diethyldithiocarbamate-ferrous oxide nanoparticles inhibit human and mouse glioblastoma stemness: aldehyde dehydrogenase 1A1 suppression and ferroptosis induction

**DOI:** 10.3389/fphar.2024.1363511

**Published:** 2024-04-24

**Authors:** Marwa M. Abu-Serie, Satoru Osuka, Lamiaa A. Heikal, Mohamed Teleb, Assem Barakat, Vikas Dudeja

**Affiliations:** ^1^ Medical Biotechnology Department, Genetic Engineering and Biotechnology Research Institute, City of Scientific Research and Technological Applications (SRTA-City), Alexandria, Egypt; ^2^ Department of Neurosurgery, School of Medicine and O’Neal Comprehensive Cancer Center, University of Alabama at Birmingham (UAB), Birmingham, AL, United States; ^3^ Department of Pharmaceutics, Faculty of Pharmacy, Alexandria University, Alexandria, Egypt; ^4^ Department of Pharmaceutical Chemistry, Faculty of Pharmacy, Alexandria University, Alexandria, Egypt; ^5^ Department of Chemistry, College of Science, King Saud University, Riyadh, Saudi Arabia; ^6^ Division of Surgical Oncology, Department of Surgery, University of Alabama at Birmingham (UAB), Birmingham, AL, United States

**Keywords:** glioblastoma stemness, chemoresistance, radioresistance, diethyldithiocarbamate-ferrous oxide nanoparticles, aldehyde dehydrogenase 1A1 inhibition, ferroptosis

## Abstract

The development of effective therapy for eradicating glioblastoma stem cells remains a major challenge due to their aggressive growth, chemoresistance and radioresistance which are mainly conferred by aldehyde dehydrogenase (ALDH)1A1. The latter is the main stemness mediator via enhancing signaling pathways of Wnt/β-catenin, phosphatidylinositol 3-kinase/AKT, and hypoxia. Furthermore, ALDH1A1 mediates therapeutic resistance by inactivating drugs, stimulating the expression of drug efflux transporters, and detoxifying reactive radical species, thereby apoptosis arresting. Recent reports disclosed the potent and broad-spectrum anticancer activities of the unique nanocomplexes of diethyldithiocarbamate (DE, ALDH1A1 inhibitor) with ferrous oxide nanoparticles (FeO NPs) mainly conferred by inducing lipid peroxidation-dependent non-apoptotic pathways (iron accumulation-triggered ferroptosis), was reported. Accordingly, the anti-stemness activity of nanocomplexes (DE-FeO NPs) was investigated against human and mouse glioma stem cells (GSCs) and radioresistant GSCs (GSCs-RR). DE-FeO NPs exhibited the strongest growth inhibition effect on the treated human GSCs (MGG18 and JX39P), mouse GSCs (GS and PDGF-GSC) and their radioresistant cells (IC_50_ ≤ 70 and 161 μg/mL, respectively). DE-FeO NPs also revealed a higher inhibitory impact than standard chemotherapy (temozolomide, TMZ) on self-renewal, cancer repopulation, chemoresistance, and radioresistance potentials. Besides, DE-FeO NPs surpassed TMZ regarding the effect on relative expression of all studied stemness genes, as well as relative p-AKT/AKT ratio in the treated MGG18, GS and their radioresistant (MGG18-RR and GS-RR). This potent anti-stemness influence is primarily attributed to ALDH1A1 inhibition and ferroptosis induction, as confirmed by significant elevation of cellular reactive oxygen species and lipid peroxidation with significant depletion of glutathione and glutathione peroxidase 4. DE-FeO NPs recorded the optimal Log*P* value for crossing the blood brain barrier. This *in vitro* novel study declared the potency of DE-FeO NPs for collapsing GSCs and GSCs-RR with improving their sensitivity to chemotherapy and radiotherapy, indicating that DE-FeO NPs may be a promising remedy for GBM. Glioma animal models will be needed for in-depth studies on its safe effectiveness.

## 1 Introduction

Glioblastoma (GBM), the most aggressive glioma subtype, is mainly attributed to glioma stem cells (GSCs), which have unique potential for self-renewal, differentiation into heterogeneous tumor cells, invasion, and treatment resistance (Alves et al., 2021; Tang et al., 2021; Sahoo et al., 2024). GSCs have a robust DNA repair system (e.g., O^6^-methylguanine-DNA methyltransferase, MGMT) that can confer resistance to the standard chemotherapeutic agent temozolomide (TMZ) by demethylation of O^6^-methylguanine, thereby eliminating the TMZ’s DNA damaging effect (Singh et al., 2021; Tang et al., 2021). There are multiple signaling pathways contributing to the aggressive features and expansion of GSCs, including Wnt/β-catenin, Hedgehog, NOTCH1, phosphatidylinositol 3-kinase (PI3K)/AKT, epidermal growth factor receptor (EGFR), and hypoxia-inducible factor (HIF1A) (Eckerdt et al., 2020; Alves et al., 2021; Tang et al., 2021). GSCs are characterized by overexpression of CD133 (PROM1), CD44, ATP-binding cassette (ABC) drug efflux transporters (ABCB1/Multidrug resistance, ABCC1/multi-drug resistance-associated protein-1, and ABCG2), NANOG, OCT-4, SOX2, ZEB1, and nestin (intermediate filament protein) as well as overactivation of aldehyde dehydrogenase (ALDH)1A. These markers are important not only for identifying GSCs, but also for mediating self-renewal and treatment resistance (Schäfer et al., 2012; Alves et al., 2021; Tang et al., 2021). Among these markers, ALDH1A1 is considered as a superior CSCs protector against toxic aldehydes-mediated oxidative damage and a major stemness regulator. ALDH1A1 activity is closely linked to functional cancer stem cells via catalyzing the biosynthesis of retinoid acid which triggers the activation of PI3K/AKT, hypoxia, NOTCH, and Wnt/β-catenin pathways. These pathways involve in stemness maintenance and therapeutic resistance via enhancing the expression of stemness transcription factors (SOX2, NANOG, OCT-4, CD44, and ZEB1) and ABC transporters. Moreover, ALDH1A1 mediates resistance to both chemotherapy and radiotherapy via the direct inactivating drugs, increasing expression of ABC transporters, and detoxifying the released reactive radicals, thus viewed as a potential therapeutic target (Schäfer et al., 2012; Ciccone et al., 2020; Uddin et al., 2020; Yang et al., 2020; Deldar Abad Paskeh et al., 2021; Poturnajova et al., 2021; Daisy Precilla et al., 2022; Wei et al., 2022).

Despite substantial improvements in GBM therapeutic methods, its treatment remains challenging in comparison to other tumor types (Stupp et al., 2009; Singh et al., 2021). Therefore, an effective remedy that targets GSCs with the goal of lowering their chemoresistance and radioresistance is critical.

Recent studies reported that nanocomplexes of diethyldithiocarbamate (DE, a metabolite of the FDA-approved drug disulfiram) with metal oxide nanoparticles (NPs) exhibited potent anticancer impact against human cancer cell lines and tumor animal models (Abu-Serie and Eltarahony, 2021; Abu-Serie and Abdelfattah, 2022; Abu-Serie, 2023; Abu-Serie and Abdelfattah, 2023; Abu-Serie, 2024). As cancer stem cells (CSCs) can resist apoptosis-dependent cell death (Safa, 2022), these efficient nanocomplexes of DE and green chemically synthesized metal oxide (Cu_4_O_3_, Cu_2_O, and FeO) NPs were able to mediated cancer cell death by inducing new nonapoptotic pathways. These novel cell death pathways including the accumulated copper-mediated cuproptosis and iron-mediated ferroptosis, lead to mitochondrial dysfunction and uncontrolled lipid peroxidation, respectively (Tang et al., 2021; Abu-Serie, 2023; Abu-Serie and Abdelfattah, 2023). Ferroptosis inducers can thus improve sensitivity of GBM to chemotherapeutic drugs (e.g., TMZ), radiation, and immunotherapy as well as targeted therapy (e.g., epidermal growth factor receptor inhibitors) and inhibit metastasis (Zhuo et al., 2022; Bo et al., 2024; Wang et al., 2024). Ferroptosis is exacerbated by the inhibition of the antioxidant glutathione system, including glutathione peroxidase (GPX)4 and its substrate glutathione (GSH) (Koppula et al., 2021; Zhuo et al., 2022). This glutathione system suppression can be mediated by DE, which is a potent inhibitor of ALDH1A1 (Abu-Serie and Abdelfattah, 2023). Previous studies found that ALDH1A1 inhibitors prevented hypoxia and stemness expression, while increasing reactive oxygen species (ROS) and CSCs’ drug sensitivity (Vassalli, 2019; Abu-Serie and Eltarahony, 2021; Abu-Serie and Abdelfattah, 2022; Abu-Serie, 2023; Abu-Serie and Abdelfattah, 2023; Abu-Serie, 2024).

Accordingly, this current study focused on investigation of the cytotoxic effect of these nanocomplexes (DE-Cu_4_O_3_ NPs, DE-Cu_2_O NPs, and DE-FeO NPs), in comparison with TMZ, on human and mouse GSCs and their radioresistant (GSCs-RR) lines. The most active nanocomplex impact on self-renewal, cancer repopulation, therapeutic resistance, stemness gene expression, p-AKT, and ALDH1A1 activity was evaluated for unveiling its anti-stemness mechanisms.

## 2 Materials and methods

### 2.1 Materials

Copper chloride, copper nitrate, vitamin C, 3-(4,5-dimethylthiazol-2-yl)-2,5-diphenyltetrazolium bromide (MTT), DMSO, heparan sulfate, primers, ALDH activity colorimetric assay kit (Cat#MAK082), thiobarbituric acid, all trans-retinal, Ellman (5,5′-dithio-bis-2(nitro benzoic acid) reagent, GSH, and malondialdehyde tetrabutylammonium salt, were obtained from Sigma-Aldrich (Saint Louis, MO, US). DE, methoxynitrosulfophenyl-tetrazolium carboxanilide (XTT), TMZ were purchased from Acros Organics (Morris Plains, NJ, US), Invitrogen (Waltham, MA, US), and MedChemExpress (NJ, United States of America), respectively. DMEM/F12 and fetal bovine serum was from Gibco (Grand Island, NY, US). B27-mius vitamin A was supplied from Invitrogen (Waltham, MA, US), whereas human recombinant FGF basic (Cat#100-18B) and human recombinant EGF (Cat#AF-100-15) were from PeproTech (Cedarbrook Drive Cranbury, NJ, US). RNeasy MinElute Cleanup Kit, High-Capacity cDNA Reverse Transcription kit, and chemiluminescent substrate were obtained from Thermo Fischer Scientific (Waltham, MA, US), while Light Cycler 480 SYBR green kit were from Roche Diagnostics (Mannheim, Germany). protein assay dye reagent concentrate, Mini-PROTEAN TGX™ gel, and Protein Assay Dye Reagent Concentrate were from Bio-Rad (Hercules, CA, US). p-AKT (rabbit monoclonal antibody (Thr308), AKT, and anti-rabbit IgG were supplied from Cell Signaling Technology (Danvers, MA, US). HRP-β-actin (Cat# ab49900 [AC-15]) and ROS assay kit were purchased from Abcam (Cambridge, MA, US).

### 2.2 Methods

#### 2.2.1 Preparation of the characterized metal oxide NPs and nanocombination with DE

**FIGURE 1:**
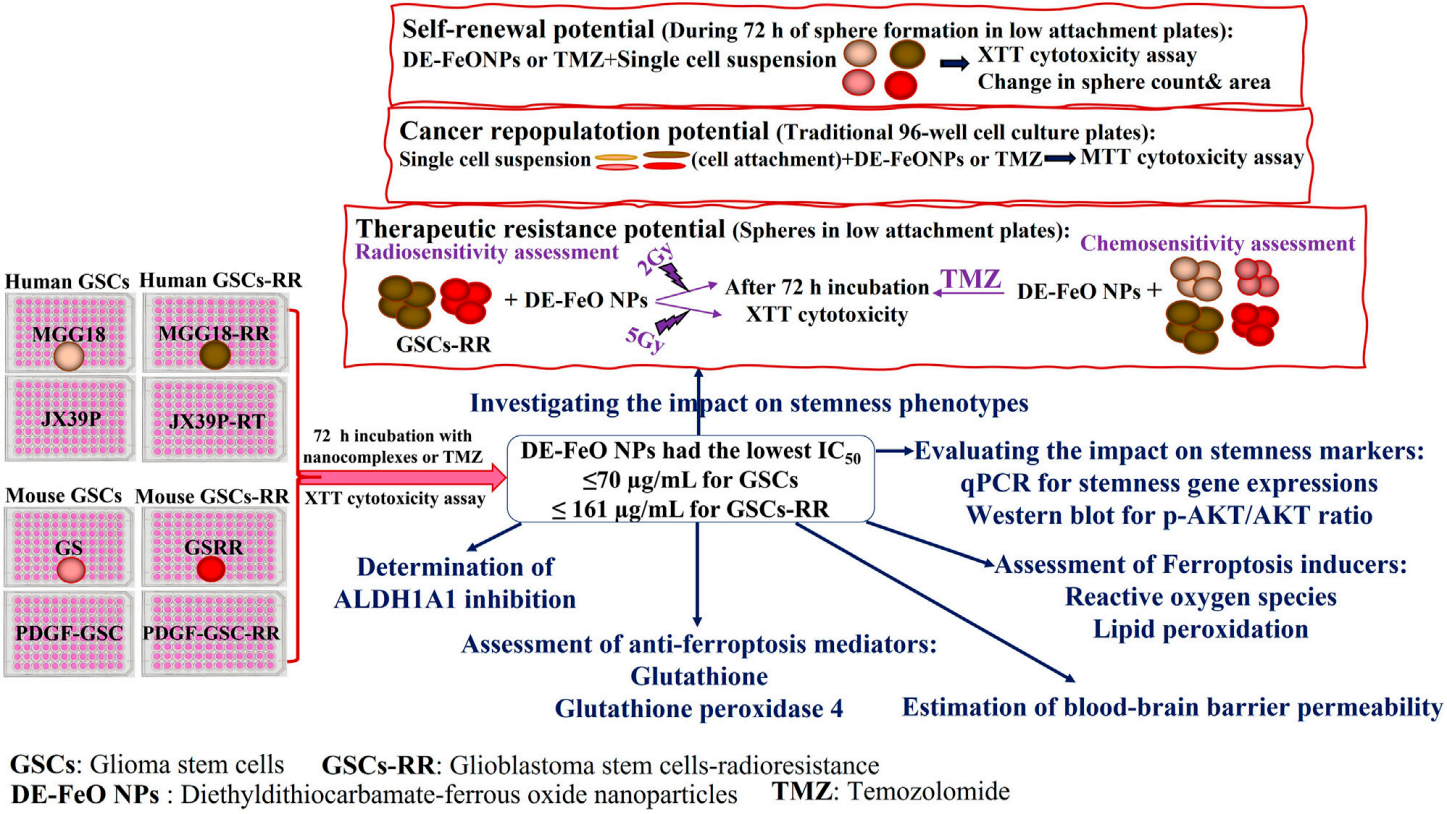
Experimental design.

Copper oxide NPs (Cu_4_O_3_ and Cu_2_O) were prepared by incubating chitosan and vitamin C with copper copper chloride and copper nitrate, respectively (Abu-Serie and Eltarahony, 2021; Abu-Serie and Abdelfattah, 2023), whereas ferrous oxide NPs were prepared by adding iron nitrate and vitamin C to NaOH (Abu-Serie and Abdelfattah, 2022). As mentioned in these our recent studies, Cu_4_O_3_ NPs, Cu_2_O NPs, and FeO NPs with sizes of 112.5, 111.6, and 38.8 nm, respectively, were characterized by X-ray diffractometer, energy dispersive X-ray analysis, and electron microscopes. These metal oxide NPs were mixed with DE, at a ratio of 1:10, forming nanocomplexes of DE-Cu_4_O_3_ NPs, DE-Cu_2_O NPs, and DE-FeO NPs (156.5, 148.1, and 157.8 nm, respectively). The identity of the pure chelated nanoformulation between DE and FeO NPs was confirmed by FTIR spectra (Abu-Serie and Abdelfattah, 2022). For the subsequent experiments, all NPs and DE were dissolved in GSCs’culture medium. The experimental design of this current study is demonstrated in Figure 1.

#### 2.2.2 Cytotoxicity assay using human and mouse GSCs and GSCs-RR

##### 2.2.2.1 Cell culture

Human MGG18 cells were provided by Dr. Hiroaki Wakimoto (Harvard Medical School, Boston, MA, US) and established as previously described (Wakimoto et al., 2009; Wakimoto et al., 2012). JX39P cells were provided by Dr. Christopher D. Willey (University of Alabama at Birmingham, AL, US), and generated as previously reported (Stackhouse et al., 2022). Mouse GS cells were isolated as recently mentioned (Osuka et al., 2021). Platelet derived growth factor-induced glioma stem cells (PDGF-GSC) were provided by Dr. Dolores Hambardzumyan (Icahn School of Medicine at Mount Sinai, NY, US) and established as demonstrated previously (Charles et al., 2010).

Radioresistant GSCs were established as shown in previous study (Osuka et al., 2021). Briefly, we performed repeated irradiation (2 or 5 Gy) to mouse GSCs (GS and PDGF-GSC) and human GSCs (MGG18), every three or 4 days *in vitro*. The culture medium was replaced, and the cells were passaged as appropriate. After repeated irradiation, we established radioresistant GSCs, MGG18-RR (2Gy x 9 doses), GSRR (5Gy x 12) and PDGF-GSC-RR (5 Gy x 8). JX39-RT is a radioresistant patient-derived xenograft (PDX) cells established by Dr. Christopher D. Willey (University of Alabama at Birmingham, AL, US). Subcutaneous PDX tumors were exposed to 2 Gy of irradiation three times per week for 2 weeks (total 12 Gy) and allowed to regrow before being passaged to a new set of mice. This process was repeated up to six times to establish radiation-resistant PDX models.

All above-mentioned GSCs were cultured in DMEM/F12 supplemented with 10 mL B27-mius vitamin A, 20 ng/mL human recombinant FGF basic, 20 ng/mL human recombinant EGF, and 200 ng/mL heparan sulfate.

##### 2.2.2.2 XTT cell proliferation assay

All GSCs (MGG18, JX39P, GS, PDGF-GSC, MGG18-RR, JX39P-RT, GSRR, PDGF-GSC-RR) were trypsinized to obtain single cell suspensions. Then, these cells were seeded (3,000 cells/well and 6,000 cells/well for GSCs and their corresponding GSCs-RR, respectively) in 96 well ultra-low attachment sterile plates. After 72 h of sphere formation, serial concentrations (300–9.375 μg/mL) of the standard chemotherapy (TMZ), DE, metal oxide NPs, and DE-Cu_4_O_3_ NPs, DE-Cu_2_O NPs, and DE-FeO NPs were added. After another 3 days of incubation in 5% CO_2_ incubator (37°C), 1 mg/mL of XTT was incubated with the untreated and treated spheres for 4 h. Then, absorbances of the formed formazan (an orange-red colored product of active metabolite spheres) were measured at 450 nm using SpectraMax microplate reader (Marshall Scientific, Hampton, NH, US) to estimate the growth inhibition percentages relative to the untreated GSCs. Subsequently, the inhibitory concentration at which 50% growth inhibition (IC_50_) was calculated by GraphPad Prism 9. Furthermore, morphological changes in the treated spheres and the untreated spheres were recorded using phase contrast inverted microscope (Fisher Scientific, Waltham, MA, US). Additionally, fluorescence microscope (Life technologies, Carlsbad, CA, US) was used to investigate mouse GSCs and GSCs-RR that had been labeled with a fluorescence reporter (mCherry).

Among human and mouse GSCs, MGG18 and GS were selected for further experiments owing to their aggressive growth behavior compared to JX39P and PDGF-GSC. In all the following assays, the most effective formula (DE-FeO NPs) and TMZ were used at doses of 70 μg/mL for GSCs (MGG18 and GS) and 161 μg/mL for GSCs-RRs (MGG18-RR and GSRR).

#### 2.2.3 Evaluation of the impact on stemness phenotypes

##### 2.2.3.1 Self-renewal capability

The single cell suspensions of MGG18, GS, MGG18-RR, and GSRR were seeded in low-attachment 96 well plates and incubated with serial concentrations of DE-FeO NPs or TMZ during 72 h of sphere formation in 5% CO_2_ incubator. Then, XTT was added and absorbance was measured at 450 nm to estimate the efficiency of sphere formation in the presence of nanocomplex and chemotherapy in relative to the untreated wells. Furthermore, IC_50_ value, at which 50% inhibition of self-renewal, was estimated by GraphPad Prism 9, for comparing between nanocomplex and chemotherapy (Abu-Serie et al., 2021). Moreover, the formed spheres in the treated wells were counted and their areas were measured by ImageJ software in relation to the untreated formed spheres. Additionally, the morphology of these formed spheres was documented by phase contrast (Fisher Scientific, Waltham, MA, US) and fluorescence microscopes (Life technologies, Carlsbad, CA, US).

##### 2.2.3.2 Cancer repopulation potential

The single cell suspensions of GSCs (MGG18 or GS) were cultured in 10% fetal bovine serum-supplemented DMEM/F12 medium and seeded in traditional 96-well cell culture plates. After cell attachment, serial concentrations of DE-FeO NPs or TMZ were added. After 3 days, 20 µL MTT (5 mg/mL) was incubated for 4 h, then discarded, and 100 µL DMSO was added followed by measuring absorbance at 590 nm (SpectraMax microplate reader, Marshall Scientific, Hampton, NH, US). The growth inhibition percentage was estimated relative to the untreated attached cancer cells and IC_50_ was calculated by GraphPad Prism 9 (Abu-Serie et al., 2021). Furthermore, the morphological alterations of these cancer cells were observed using phase contrast (Fisher Scientific, Waltham, MA, US) and fluorescence microscopes (Life technologies, Carlsbad, CA, US).

##### 2.2.3.3 Resistance response of GSCs-RR to chemotherapy and radiotherapy

After preincubation of MGG18-RR and GSRR with serial concentrations of DE-FeO NPs for 24 h, these spheres were exposed to resistance dose of TMZ (10 μg/mL) or resistance irradiation doses (2 Gy and 5 Gy). After 3 days, cell viability was measured by adding XTT and sphere morphology was recorded by phase contrast (Fisher Scientific, Waltham, MA, US) and fluorescence microscopes (Life technologies, Carlsbad, CA, US). The fold increment in sensitivity to chemotherapy and radiotherapy was calculated relative to TMZ-treated and irradiated spheres, respectively.

#### 2.2.4 qPCR assessment for key stemness genes

RNeasy MinElute Cleanup Kit was used to extract RNA from the untreated and treated GSCs and GSCs-RR. After quantifying RNA by NanoDrop spectrophotometer (Thermo Fischer Scientific, Waltham, MA, US) and cDNA synthesis using High-Capacity cDNA Reverse Transcription kit, qPCR was performed using Light Cycler 480 SYBR green kit and primers “final concentration 500 nM”. Human and mouse primer sequences and their pearson’s coefficient and efficiency were shown in Supplementary Tables S1, S2. The qPCR protocol was initiated at 95°C for 5 min, followed by 40 cycles of 95°C (20 s) and 60°C (1 min).

#### 2.2.5 Western blot analysis of p-AKT and AKT protein levels

For determining p-AKT level relative to total AKT, Western blot technique was used. Briefly, cellular proteins of the untreated and treated spheres were extracted, quantified by protein assay dye reagent concentrate, boiled in loading buffer, and separated using 4%–20% Mini-PROTEAN TGX™ gel. Then, proteins were transferred to nitrocellulose membrane, blocked with 5% bovine serum albumin, washed, and incubated with 1:1,000 p-AKT (rabbit monoclonal antibody Thr 308) or AKT overnight at 4°C. After that, the membrane was washed, incubated 1: 2000 secondary antibody (anti-rabbit IgG) for 1 h, washed, and chemiluminescent substrate was added, then bands were documented using chemiluminescent imaging system (ImageQuant™ LAS 500, GE Healthcare Bio-Sciences, Piscataway, NJ, US). For housekeeping protein, the membrane was washed, and incubated with 1:1000 HRP-β-actin for 1 h, washed, and the substrate was added for visualizing its bands using chemiluminescent imaging system (ImageQuant™ LAS 500, GE Healthcare Bio-Sciences, Piscataway, NJ, US).

#### 2.2.6 Determination of ALDH1A1 inhibition

For determination of ALDH1A1 activity, ALDH activity colorimetric assay kit was used with all trans-retinal as substrate. Following 72 h of treatment of the above-mentioned MGG18, GSRR, and their GSCs-RR with DE-FeO NPs or TMZ, these spheres were lysed in ALDH assay buffer, centrifuged, and the supernatants were harvested for incubation with ALDH substrate mix and retinal. The change in the absorbance was recorded after 10 min at 450 nm using microplate reader (Marshall Scientific, Hampton, NH, US), and the activity was calculated using the NADH standard curve. Then, the inhibition percentage in ALDH1A1 activity was estimated relative to the untreated spheres.

#### 2.2.7 Determination of GSH level and GPX4 activity

Cellular GSH and GPX4 were determined in cell lysates of the untreated and treated cells. GSH level was quantified using Ellman (5,5′-dithio-bis-2(nitro benzoic acid) reagent as previously reported (Ellman, 1959; Abu-Serie and Abdelfattah, 2022). GPX4 activity was detected as described in previous studies (Kernstock and Girotti, 2008; Abu-Serie, 2023). Then, GPX4 inhibition percentage in the treated spheres was estimated relative to the untreated spheres.

#### 2.2.8 Quantification of cellular contents of ROS and lipid peroxidation

Reactive oxygen species level was detected in the untreated and treated spheres using cellular ROS assay kit. After 72 h of treatment with DE-FeO NPs or TMZ, all spheres were resuspended in phenol red-free DMEM/F12 culture medium and incubated with dichlorodihydrofluorescein diacetate (DCF-DA) for 60 min in 5% CO_2_ incubator (37°C). The fluorescence intensity of DCF (equivalent to cellular ROS content) was measured at 485 nm (excitation) and 535 nm (emission) using fluorescence microplate reader (Marshall Scientific, Hampton, NH, US). The level of ROS was estimated using standard curve of *t*-butyl hydroperoxide. The latter values were normalized to cellular protein content which was detected by Protein Assay Dye Reagent Concentrate.

Briefly, lipid peroxidation content was detected by incubation of supernatant of the untreated and treated spheres with an equal volume of 0.67% thiobarbituric acid in 95°C for 60 min (Innes et al., 1988). Then, plates were centrifuged and the supernatants were measured at 532 nm. The concentration of lipid peroxides was calculated using standard curve of malondialdehyde and normalized to the cellular protein level.

#### 2.2.9 Experimental determination of blood-brain barrier permeability (Log*P*)

The reference substances, benzoic acid, toluene, anthraquinone and benzyl benzoate were dissolved in HPLC grade methanol. Their retention time on RP-C18 column was then determined. The HPLC analysis employed reversed-phase C18 column (Agilent HC-C18(2), 5 μm, 150 mm × 4.6 mm, Agilent Technologies, Inc., Santa Clara, CA, US) using photodiode detector (Agilent 1,260 Infinity DAD, Agilent Technologies, Inc., Santa Clara, CA, US). For analysis, an isocratic mobile phase of 3:1 (v/v) methanol and water was employed. A volume of 20 μL (Agilent 1,260 Infinity High S61 performance autosampler) was injected with a flow rate of 0.6 mL/min (Agilent 1,260 Infinity Quaternary Pump). Temperature of the column oven was kept at 25°C (Agilent 1,260 Infinity Thermostatic Column Compartment). A calibration curve of Log k versus Log*P* of the reference compounds was constructed to obtain its regression equation (Hansch and Leo, 1980). The most active nanocomplex (DE-FeO NPs) was dissolved in the mobile phase that was injected into the column. The partition coefficient of the tested nanocomplex was calculated from interpolating its capacity factor value in the calibration curve regression equation. Log*P* of this nanocomplex was calculated by extrapolating its t ratio using regression equation.

#### 2.2.10 Statistical analysis

Data was presented as mean ± standard error of mean (SEM). The obtained data (n = 6–9) was analyzed using *t*-test and one-way analysis of variance (ANOVA) multiple comparison with Tukey’s *post hoc* test in GraphPad Prism 9.3.1. The statistical significance levels were set at *p* ≤ 0.05*, ≤0.01**, and ≤0.001***. Normal distribution and homogeneity were checked using D’Agostino-Pearson omnibus normality test (GraphPad Prism 9.3.1) and homogeneity of variance test (SPSS Statistics 27), respectively.

## 3 Results

### 3.1 Growth inhibition potentials on human and mouse GSCs and GSCs-RR

**FIGURE 2:**
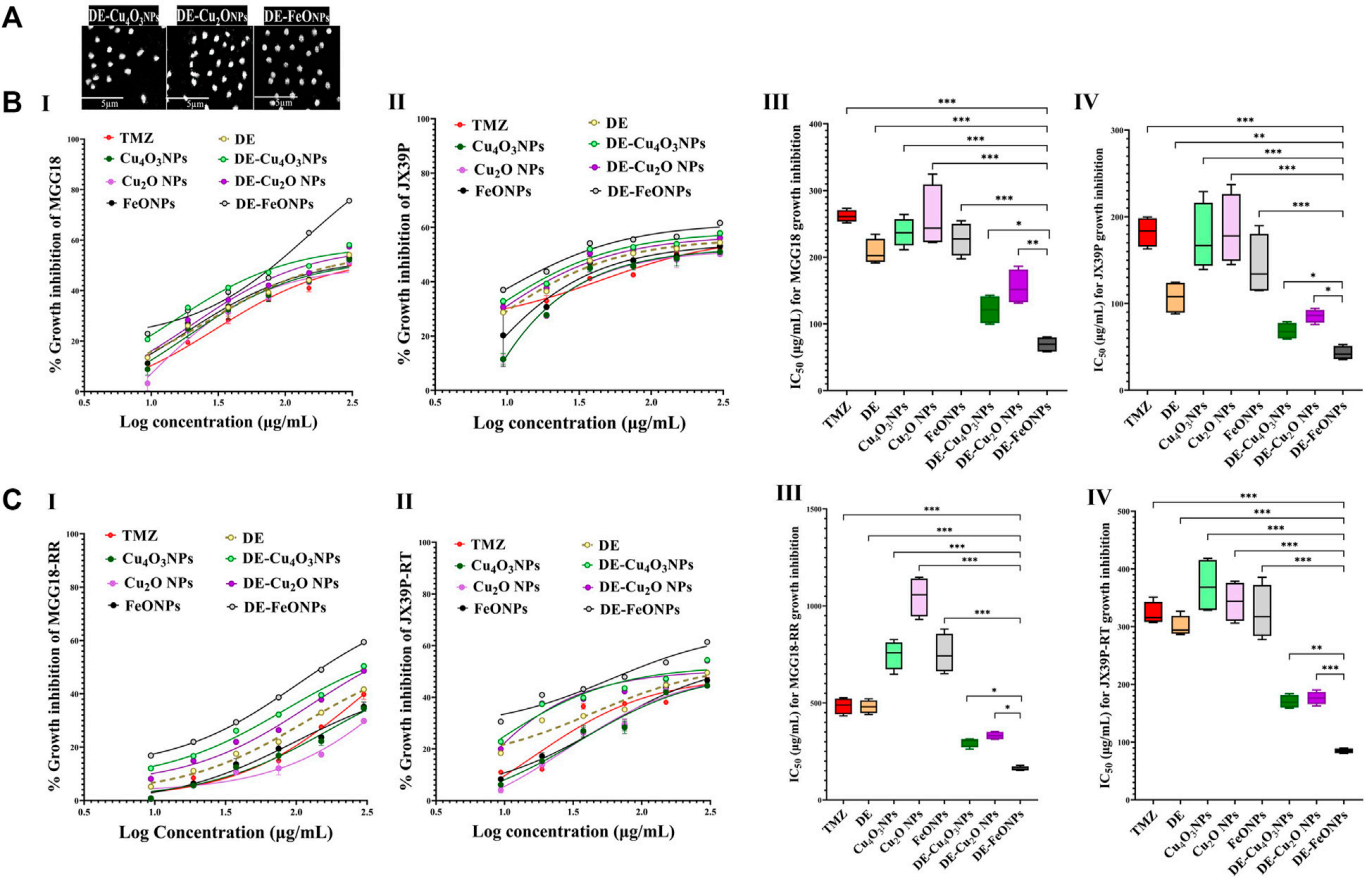
Morphology of the studied nanocomplexes and cytotoxicity on human glioma stem cells (GSCs) and corresponding radioresistance (GSCs-RR). **(A)** Scanning electron microscope images of diethyldithiocarbamate (DE)-Cu_4_O_3_ NPs, DE-Cu_2_O NPs, and DE-FeO NPs (Magnification ×5,000). **(B)** Growth inhibition potentials of standard chemotherapy (temozolomide, TMZ), DE, metal oxides (Cu_4_O_3_ NPs, Cu_2_O NPs, and FeO NPs), and nanocomplexes (DE-Cu_4_O_3_ NPs, DE-Cu_2_O NPs, and DE-FeO NPs) as shown by (I,II) dose response curves and (III,IV) half maximal inhibitory concentration (IC_50_) values for MGG18 and JX39P, respectively. **(C)** Growth inhibition potentials of these above-mentioned compounds on MGG18-RR and JX39P-RT as demonstrated by (I,II) dose response curves and (III,IV) IC_50_. Data are demonstrated as mean ± SEM. DE-FeO NPs nanocomplex was compared to other compounds. Values are considered statistically significant at *p* < 0.05*, <0.01**, and <0.001***.

**FIGURE 3:**
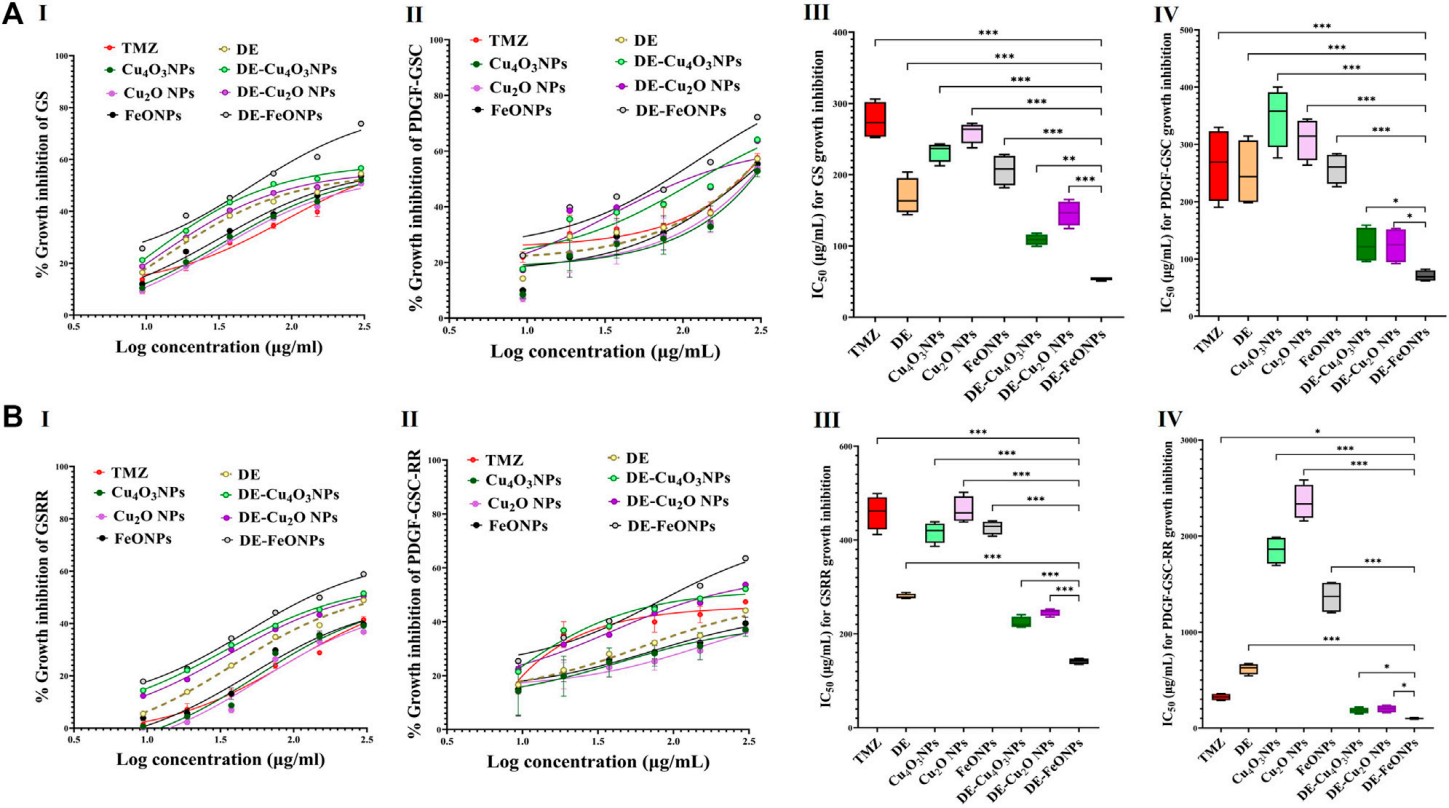
Cytotoxicity on mouse glioma stem cells (GSCs) and correlative radioresistance GSCs-RR. **(A)** Growth inhibition impacts of standard chemotherapy (temozolomide, TMZ), diethyldithiocarbamate (DE), metal oxides nanoparticles (Cu_4_O_3_ NPs, Cu_2_O NPs, and FeO NPs), and nanocomplexes (DE-Cu_4_O_3_ NPs, DE-Cu_2_O NPs, and DE-FeO NPs), as illustrated by (I,II) dose response curves and (III,IV) half maximal inhibitory concentration (IC_50_) values for GS and platelet derived growth factor-induced glioma stem cells (PDGF-GSC), respectively. **(B)** Growth inhibition potentials of these compounds on GSRR and PDGF-GSC-RR as presented by (I,II) dose response curves and (III,IV) IC_50_. Data are demonstrated as mean ± SEM. DE-FeO NPs nanocomplex was compared to other compounds. Values are considered statistically significant at *p* < 0.05*, <0.01**, and <0.001***.

**FIGURE 4:**
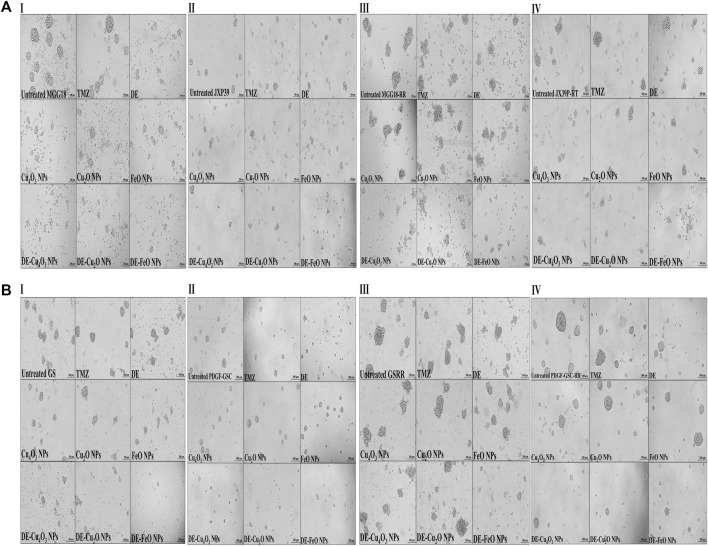
Morphological alterations of the treated human and mouse glioma stem cells (GSCs) and their radioresistance GSCs-RR, using phase contrast microscopy. **(A)** Human GSCS, including (I) MGG18, (II) JX39P, (III) MGG18-RR, and (IV) JX39P-RT and **(B)** Mouse GSCS, including (I) GS, (II) PDGF-GSC, (III) GSRR, and (IV) PDGF-GSC-RR after 72 h treatment with temozolomide (TMZ), diethyldithiocarbamate (DE), metal oxides (Cu_4_O_3_ NPs, Cu_2_O NPs, and FeO NPs), and nanocomplexes (DE-Cu_4_O_3_ NPs, DE-Cu_2_O NPs, and DE-FeO NPs). (Magnification ×200, scale bar 100 µm).

Herein, nanocomplexes of DE-Cu_4_O_3_ NPs, DE-Cu_2_O NPs, and DE-FeO NPs which have semi-oval or semi-circular shapes (Figure 2A), were investigated for their cytotoxicity on GSCs and GSCs-RR in comparison with their constituents (metal oxide NPs and DE) as well as TMZ. Regarding human GSCs (MGG18 and JX39P), DE-FeONPs had the highest growth inhibition potency with the lowest half maximal inhibitory concentration (IC_50_ ≤ 70 and 43 μg/mL, respectively) compared to DE-Cu_4_O_3_ NPs, DE-Cu_2_O NPs, metal oxides, DE, and TMZ (262 and 182 μg/mL, respectively) as shown in Figure 2B (I–IV). For their corresponding GSCs-RR (MGG18-RR and JX39P-RT), DE-FeONPs exhibited the strongest cytotoxic effect with the minimum IC_50_ (142 and 85 μg/mL, respectively) compared with other two nanocomplexes, metal oxides, DE, and TMZ (485 and 322 μg/mL, respectively), as illustrated in Figure 2C (I–IV). Furthermore, this nanocomplex of DE-FeONPs revealed the lowest IC_50_ values (54, 70, 161, 103 μg/mL) compared to other tested compounds and TMZ (276, 265, 458, and 323 μg/mL) for inhibiting the growth of mouse GSCs (GS and PDGF-GSC) and GSCs-RR (GSRR and PDGF-GSC-RR), respectively, as demonstrated in Figure 3A (I–VI), Figure 3B (I–VI). Figure 4A (I–VI), Figure 4B (I–IV) declares that the morphology of the DE-FeONPs-treated human and mouse GSCs and GSCs-RR, as compared to those other treated spheres and untreated control spheres, had severely collapsed. Additionally, Supplementary Figure S1 (A–D) illustrates the obvious decrease in red fluorescence of mCherry-labeled GS, PDGF-GSC, and their correlative GSCs-RR after DE-FeONPs treatment compared to untreated and other compounds-treated mCherry-labeled GSCs and GSCs-RR.

Accordingly, the most active compound (DE-FeONPs) was chosen to study its inhibitory effect on the stemness of GSCs and GSCs-RR at its IC_50_ (70 and 161 μg/mL, respectively), in comparison with TMZ. Since MGG18 and GS, along with their correlating RR, exhibit more aggressive growth patterns than JX39P and PDGF-GSC, these GSCs were used in the subsequent assays.

### 3.2 Suppressive effect on self-renewal, cancer repopulation, and therapeutic resistance potentials

**FIGURE 5:**
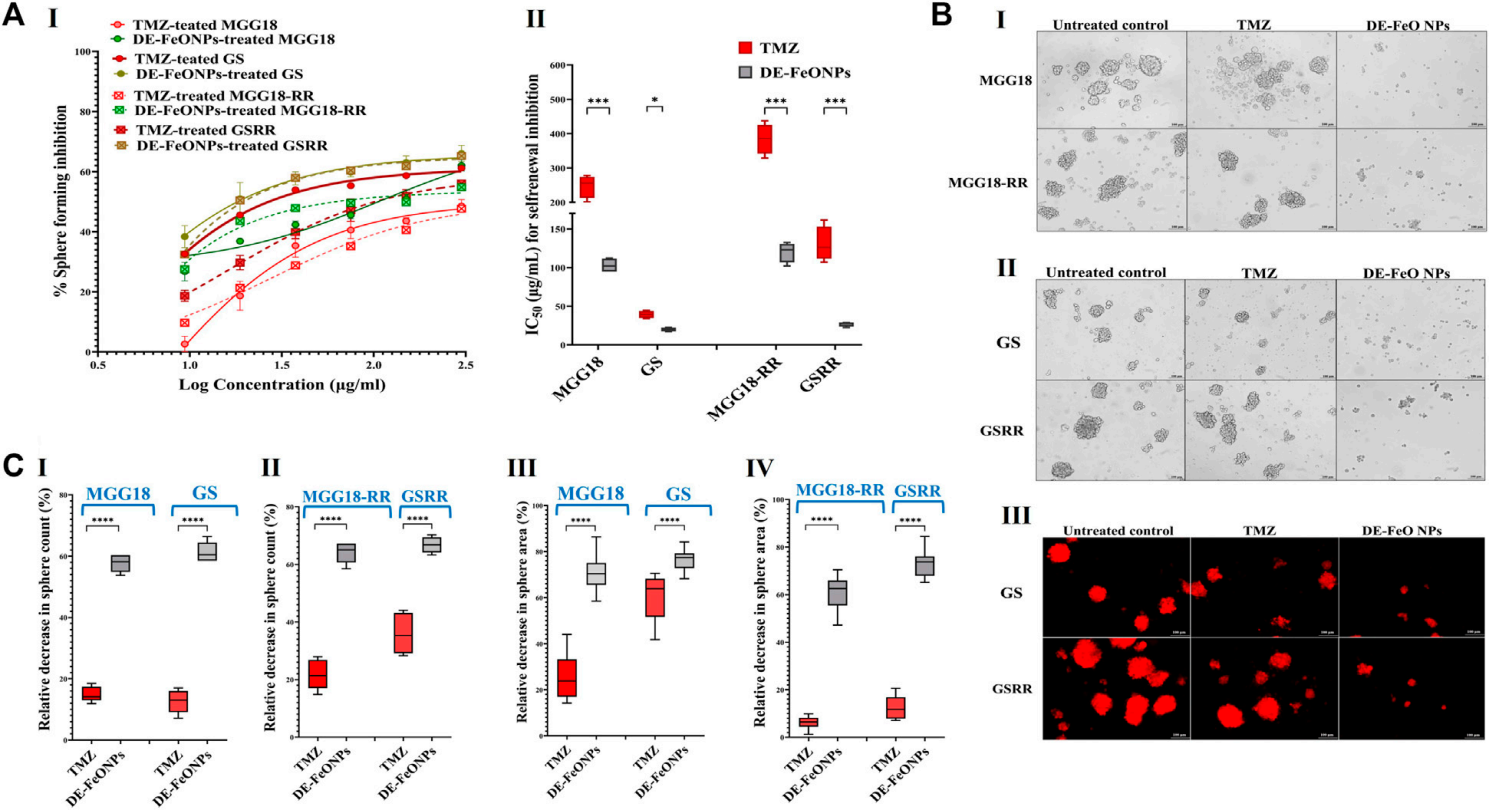
Inhibitory impacts of standard chemotherapy and the most active nanocomplex on the self-renewal potential of human and mouse glioma stem cells (GSCs) and radioresistance GSCs-RR. **(A)** Inhibition potency of temozolomide (TMZ) and diethyldithiocarbamate (DE)-FeO NPs on sphere formation of human MGG18, mouse GS, MGG18-RR, and GSRR as indicated by (I) dose response curves and (II) half maximal inhibitory concentration (IC_50_) values as well as **(B)** morphological variations of the formed spheres using (I,II) phase contrast microscopy and (III) fluorescence microscopy for mCherry-labeled GS and GSRR. **(C)** A decrease in (I,II) counts and (III,IV) area of the formed treated spheres (MGG18, GS, MGG18-RR, and GSRR, respectively) relative to the untreated control. Data are demonstrated as mean ± SEM. DE-FeO NPs nanocomplex was compared to TMZ. Values are considered statistically significant at *p* < 0.05*, <0.01**, and <0.001***.

The self-renewal inhibition was detected by incubating single cell suspensions of these aforementioned spheres with serial concentrations of the most active compound (DE-FeONPs) or TMZ for the 3 days that are required for sphere formation. Figure 5A (I, II) declares that 103, 20, 116, and 27 μg/mL of DE-FeONPs, in comparison to 248, 39, 385, and 130 μg/mL of TMZ, can inhibit 50% of the self-renewal potential of MGG18, GS, MGG18-RR, and GSRR, respectively. Figure 5B (I–II) confirms the inhibitory potency of DE-FeONPs on sphere formation in the treated human and mouse mCherry-labeled GSCs and GSCs-RR, in comparison with TMZ. As recorded, DE-FeONPs diminished the count of the generated spheres of the above-mentioned cells by 64.2%, 66.8%, 57.7%, and 61.3%, respectively, compared to 21.8%, 36%, 15%, and 12.7%, respectively, in the case of TMZ (Figure 5C (I, II)). Moreover, areas of DE-FeONPs-treated spheres were reduced by 70.7%, 76.5%, 61.2%, and 73.1%, respectively, while TMZ-treated sphere areas decreased by 25.6%, 59.8%, 6.05%, 12.5%, respectively (Figure 5C (III, IV)).

**FIGURE 6:**
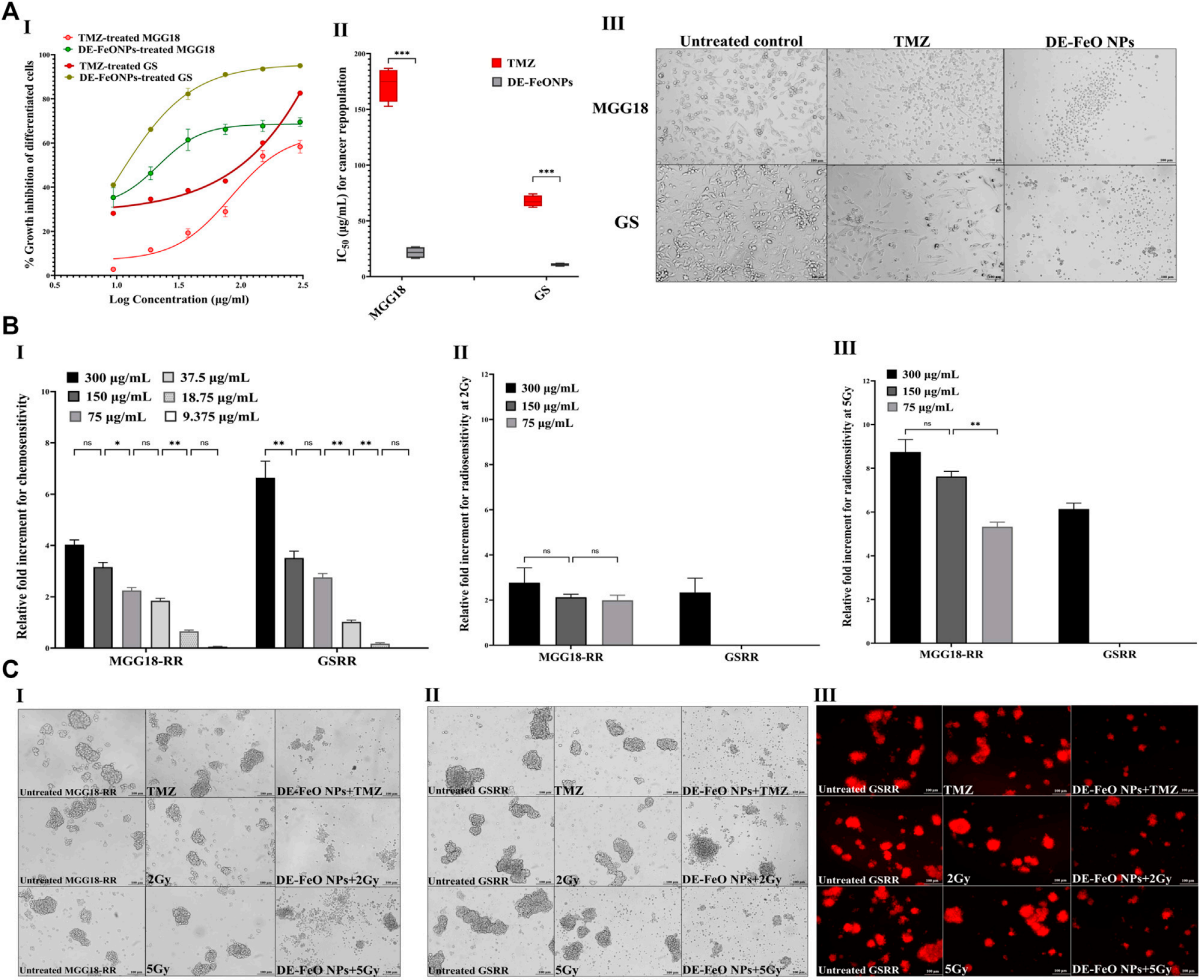
Suppressive effects of standard chemotherapy and the most active nanocomplex on cancer repopulation and therapeutic resistance potentials of human and mouse glioma stem cells (GSCs) and radioresistance GSCs-RR. **(A)** Growth inhibition effect of temozolomide (TMZ) and diethyldithiocarbamate (DE)-FeO NPs on the attached differentiated glioma cells of human MGG18 and mouse GS in terms of (I) dose response curves and (II) half maximal inhibitory concentration (IC_50_) values, as well as (III) morphological variations in the treated differentiated cancer cells using phase contrast microscopy (Magnification ×200, scale bar 100 µm). Data are demonstrated as mean ± SEM. DE-FeO NPs nanocomplex was compared to TMZ. Values are considered statistically significant at *p* < 0.05*, <0.01**, and <0.001***. **(B)** Relative fold increment in the chemosensitivity and radiosensitivity response of pretreated MGG18-RR and GSRR with DE-FeO NPs to (I) TMZ and (II,III) radiation (2Gy and 5Gy, respectively), as well as **(C)** their morphology (I) human spheres using phase contrast microscopy (Magnification ×200, scale bar 100 µm) and mouse spheres using (II) phase contrast microscopy and (III) Fluorescence microscopy (magnification ×200, scale bar 100 µm).

For cancer repopulation inhibition, single cell suspensions of MGG18 and GS were cultured in medium containing fetal bovine serum, seeded in regular cell culture plates, and then incubated with the serial dilutions of DE-FeONPs or TMZ. Cells of MGG18-RR or GSRR did not properly form an attached monolayer and proliferated as spheres in this attached condition, so MGG18 and GS only were used for this experiment. As illustrated in Figure 6A (I, II), this nanocomplex can inhibit 50% of the growth of differentiated cancer cells of MGG18 and GS at doses of 21.5 ± 2.6 and 10.76 ± 0.6 μg/mL, respectively, whereas IC_50_ values of TMZ were 172.3 ± 7.5 and 67.6 ± 2.5 μg/mL, respectively. Furthermore, Figure 6A (III) demonstrates severe collapse in the shape of DE-FeONPs-treated attached glioma cells in comparison with TMZ-treated cells and the untreated cells.

Regarding chemoresistance and radioresistance suppression, MGG18-RR and GSRR were chosen owing to their higher therapeutic resistance potency than MGG18 and GS. Prior to being exposure to resistance doses of TMZ or x-ray (2 Gy and 5 Gy), these spheres were preincubated with various concentrations of DE-FeONPs. Figure 6B I declares that DE-FeONPs increased the chemosensitivity of MGG18-RR and GSRR by about 4.0 and 6.6 folds at 300 μg/mL, 3.2 and 3.5 at 150 μg/mL, 2.2 and 2.7 at 75 μg/mL, and 1.8 and 1.0 folds at 37.5 μg/mL, respectively. Moreover, DE-FeONPs improved the radiosensitivity of MGG18-RR to 2 Gy and 5 Gy by 3 and 8.7 folds at 300 μg/mL, 2.1 and 7.6 folds at 150 μg/mL, and two and 5.3 folds at 75 μg/mL, respectively (Figure 6B (II, III)). Meanwhile, only the highest dose of DE-FeONPs (300 μg/mL) can enhance the radiosensitivity of GSRR to 2 Gy and 5 Gy by 2.1 and 6.1 folds, respectively (Figure 6B (II, III)). Figure 6C (I–IV) demonstrate an extreme change in the morphology and a drastic reduction in mCherry fluorescence of DE-FeONPs + TMZ, DE-FeONPs+2Gy, and DE-FeONPs+5Gy-GSCs-RR which preincubated with DE-FeONPs in comparison with the untreated control and TMZ-, 2Gy-, and 5Gy-exposed GSCs-RR.

### 3.3 Repressive impact on stemness genes, relative p-AKT level, and ALDH1A1 activity

**FIGURE 7:**
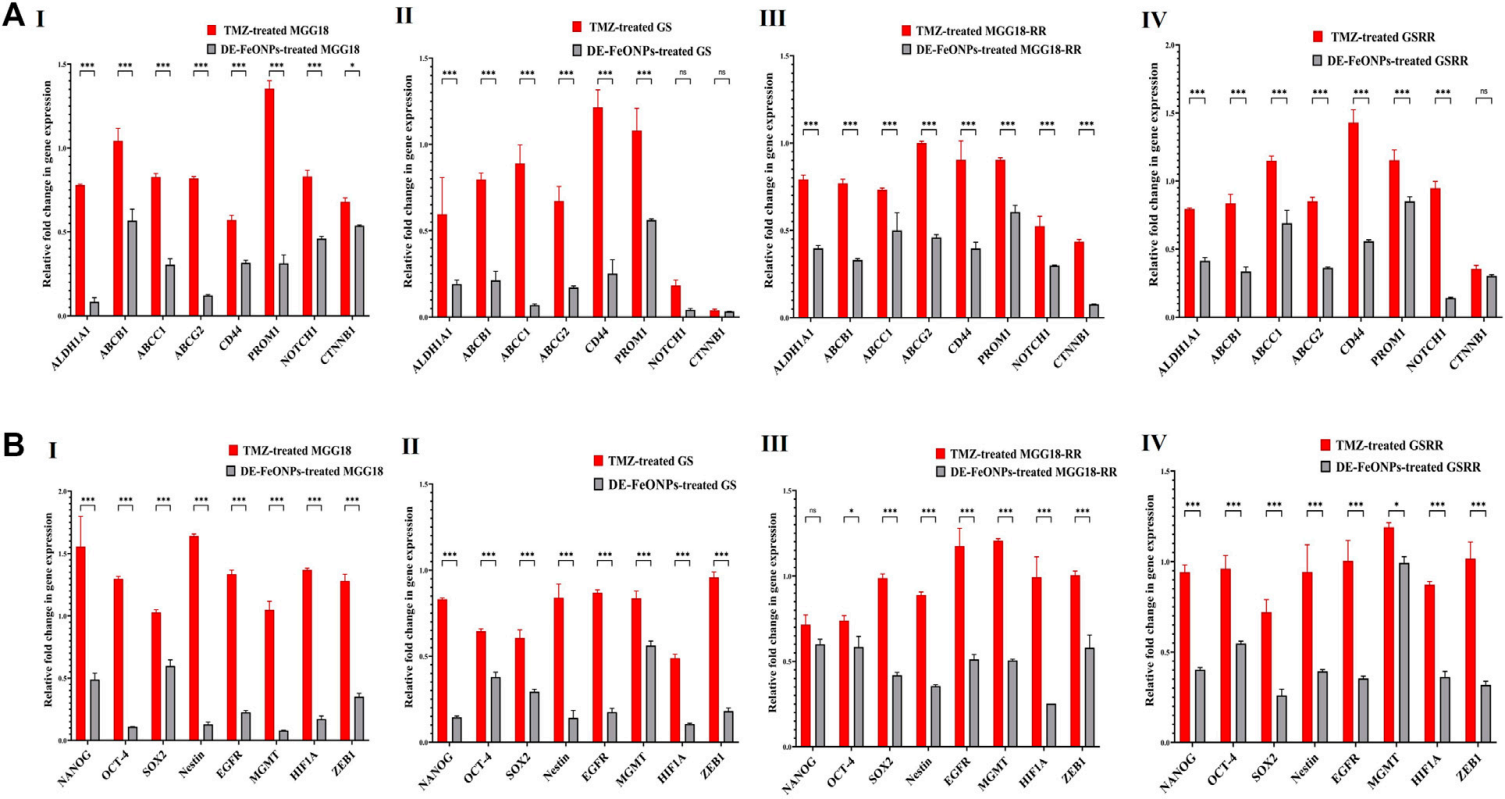
Repressive potentials of standard chemotherapy and the most active nanocomplex on stemness gene expression of human and mouse glioma stem cells (GSCs) and radioresistance GSCs-RR. Relative fold decrement in gene expression of **(A)** aldehyde dehydrogenase (ALDH)1A1, ATP-binding cassette (ABC)B1, ABCC1, ABCG2, CD44, CD133 (PROM1), NOTCH1, and β-catenin (CTNNB1) as well as **(B)** NANOG, OCT-4, SOX-2, nestin, epidermal growth factor receptor (EGFR), O^6^-methylguanine-DNA methyltransferase (MGMT), hypoxia inducing factor (HIF1A), and ZEB1 in (I) human MGG18, (II) mouse GS, (III) MGG18-RR, and (IV) GSRR after treatment with temozolomide (TMZ) and diethyldithiocarbamate (DE)-FeO NPs. Data are demonstrated as mean ± SEM. DE-FeO NPs nanocomplex was compared to TMZ. Values are considered statistically significant at *p* < 0.05*, <0.01**, and <0.001***.

As shown in Figures 7A, B, DE-FeONPs significantly downregulated the expression of the studied genes when compared to TMZ. However, no significant difference was recorded between DE-FeONPs and TMZ in the relative expression of NOTCH1 and β-catenin (CTNNB1) in the treated GS, NANOG in the treated MGG18-RR, and CTNNB1 in the treated GSRR. In the treated MGG18, DE-FeONPs suppressed the expression of ABCC1, ABCG2, ALDH1A1, CD44, PROM1, EGFR, HIF1A, MGMT, nestin, and OCT-4 by 3-12 folds and other genes by about two folds, compared to TMZ (Figures 7A, B I). DE-FeONPs reduced the expression of all mentioned genes by > 3 folds, excluding PROM1 and MGMT by 1.8 folds in the treated GS (Figures 7A, B II). Moreover, the expression of all genes was suppressed by ≥ 2 folds and CTNNB1 by 13 folds in DE-FeONPs-treated MGG18-RR, compared to TMZ-treated MGG18-RR (Figures 7A, B III). In DE-FeONPs-treated GSRR spheres, their gene expressions were inhibited by ≥ 2 folds, excluding ABCC1, PROM1, and MGMT expressions were ≤1.5 folds, compared to TMZ-treated GSRR [Figures 7A, B (IV)].

**FIGURE 8:**
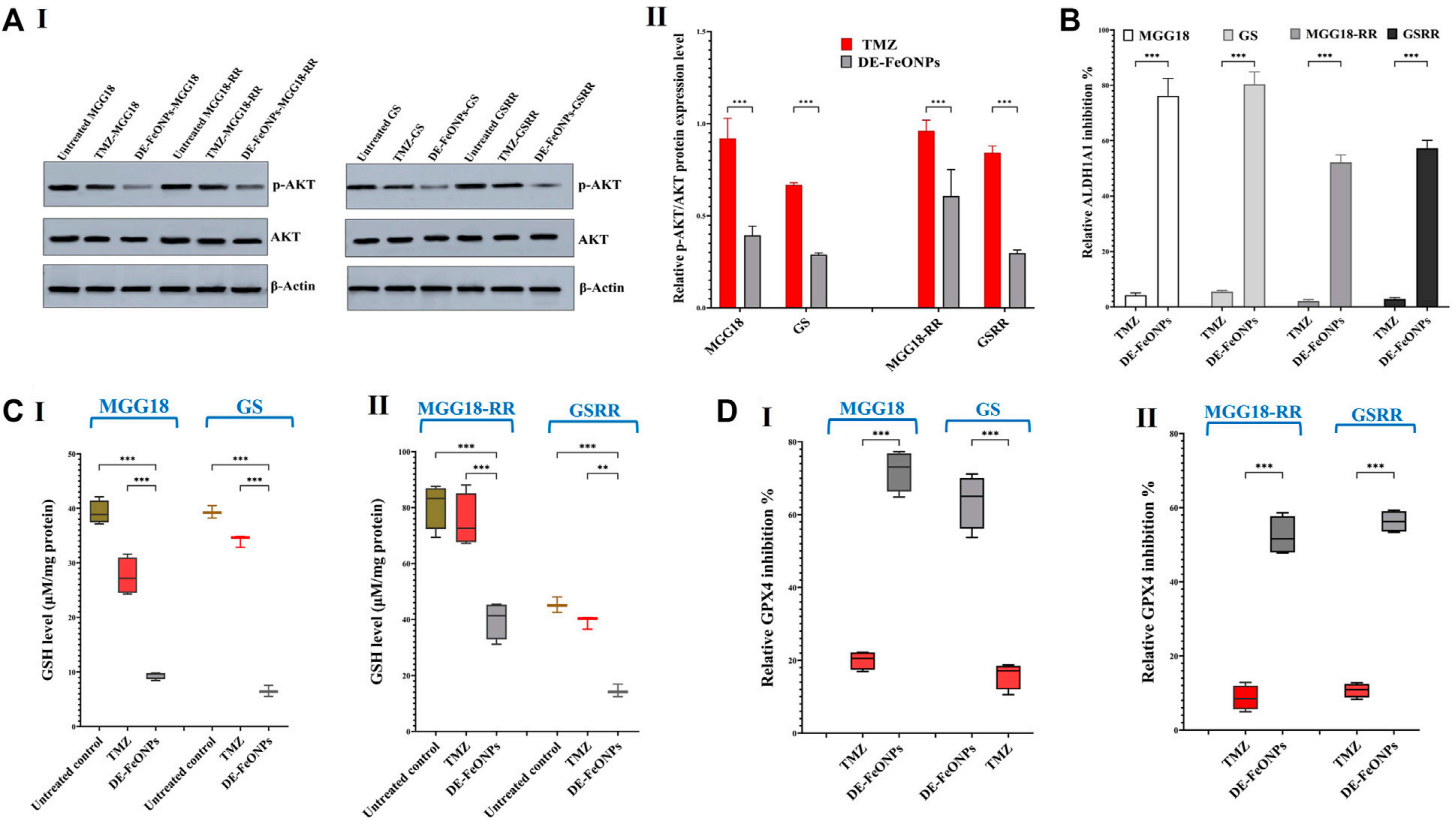
Quelling effects of standard chemotherapy and the most active nanocomplex on p-AKT/AKT, aldehyde dehydrogenase (ALDH)1A activity, glutathione (GSH) level, and glutathione peroxidase (GPX) four activity in human and mouse glioma cells (GSCs) and radioresistance GSCs-RR. **(A)** Western blot analysis of p-Akt in the untreated and temozolomide (TMZ) and diethyldithiocarbamate (DE)-FeO NPs-treated human MGG18, mouse GS, MGG18-RR, and GSRR as demonstrated by (I) Western blotting images and (II) relative ratio of p-AKT to AKT expression level. **(B)** ALDH1A1 inhibition % in the above-mentioned treated spheres relative to the untreated spheres. **(C)** Cellular level of GSH and **(D)** relative GPX4 inhibition % after treatment with TMZ and DE-FeO NPs in (I) the treated MGG18 and GS as well as (II) their corresponding radioresistance cells. Data are demonstrated as mean ± SEM. DE-FeO NPs nanocomplex was compared to TMZ and the untreated control. Values are considered statistically significant at *p* < 0.05*, <0.01**, and <0.001***.

More importantly, Western blot analysis of relative expression of p-AKT/AKT revealed that DE-FeONPs lowered significantly relative p-AKT protein level by 2.56, 1.71, 3.46, and 3.37 folds, compared to 1.10, 1.04, 1.50, and 1.19 folds in TMZ-treated MGG18, MGG18-RR, GS, and GSRR, respectively (Figure 8A (I, II)).

It is worth mentioning that DE-FeONPs inhibited extremely ALDH1A1 activity in the treated MGG18, MGG18-RR, GS, and GSRR by 76.2 ± 3.6, 80.4 ± 2.5, 52.1 ± 1.6, and 57.3% ± 1.6%, respectively, whilst TMZ suppressed it by only ≤5.5% (Figure 8B).

### 3.4 Ferroptosis induction with alteration in cellular redox status

In terms of anti-ferroptotic mediators (antioxidants), DE-FeONPs significantly diminished GSH level by 76.1, 83.5, 50.7, and 67.9%, respectively, compared to 29.8, 13.2, 7.1, and 13.3%, respectively, in TMZ-treated MGG18, GS, MGGR18-RR, and GSRR (Figure 8C (I, II)). Furthermore, GPX4 activity was significantly inhibited (63.8, 72.5, 50.3, and 55.6%) in DE-FeONPs-treated MGG18, GS, MGGR18-RR, and GSRR, respectively, when compared to TMZ-treated spheres (14.9, 21.1, 8.5, and 10.8%) as shown in Figure 8D (I, II).

**FIGURE 9:**
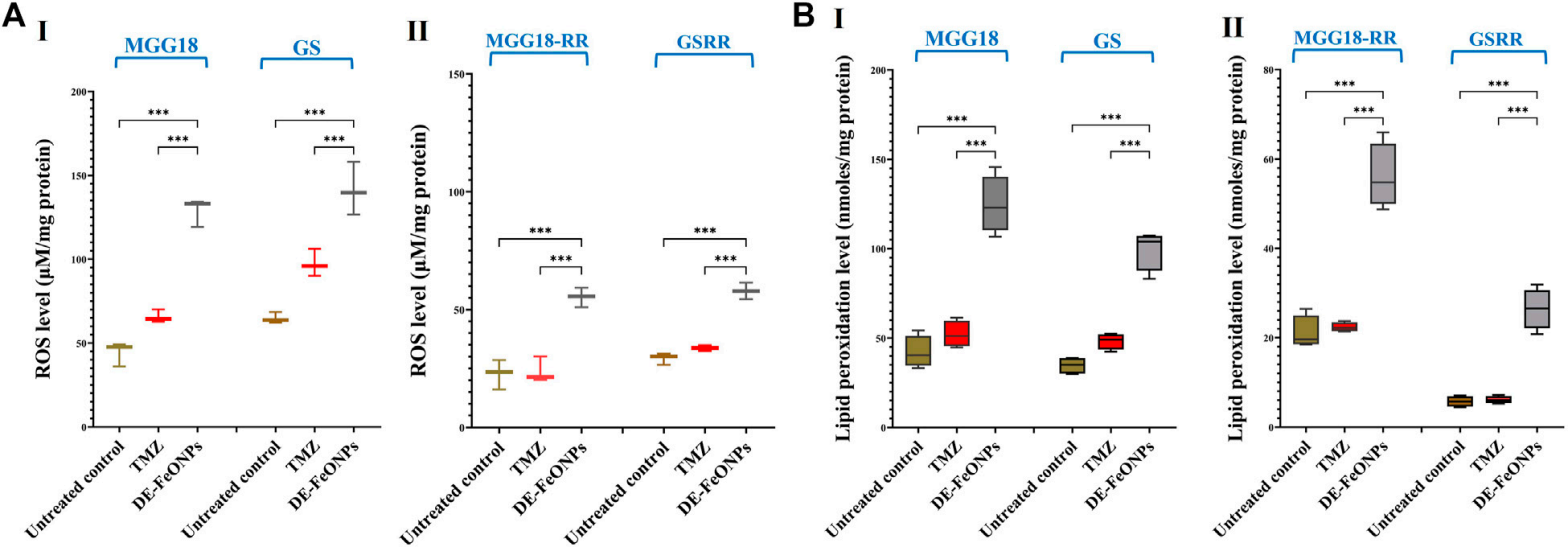
Elevation in the cellular levels of reactive oxygen species (ROS) and lipid peroxidation in standard chemotherapy and the most active nanocomplex-treated human and mouse glioma stem cells (GSCs) and radioresistance GSCs-RR. **(A)** ROS levels in (I) human MGG18, mouse GS, (II) MGG18-RR, and GSRR as well as **(B)** lipid peroxidation inhibition % in (I) human MGG18, mouse GS, (II) MGG18-RR, and GSRR after treatment with temozolomide (TMZ) and diethyldithiocarbamate (DE)-FeO NPs. Data are demonstrated as mean ± SEM. DE-FeO NPs nanocomplex was compared to TMZ and the untreated control. Values are considered statistically significant at *p* < 0.05*, <0.01**, and <0.001***.

In DE-FeONPs-treated MGG18 and GS, ROS levels were increased by 2.9 and 2.2 folds, respectively, compared to about 1.5 folds in the case of TMZ-treated spheres. In the treated MGGR18-RR and GSRR, only DE-FeONPs, not TMZ, can increase ROS levels by 2.4 and 2.0 folds, respectively (Figure 9A (I, II)). The main ferroptosis marker (lipid peroxidation) was elevated by 2.9, 2.9, 2.7, and 5.0 folds in DE-FeONPs-treated MGG18, GS, MGGR18-RR, and GSRR, respectively, versus 1.1, 1.5, 1.0, and 1.2 folds, respectively, for TMZ-treated spheres (Figure 9B (I, II)).

### 3.5 Prediction of blood-brain barrier penetration

Experimental determination of LogP for DE-FeONPs showed that the nanocomplex recorded LogP = 2.105, fulfilling the optimal LogP value for blood-brain barrier penetration (range of 1.5-2.7, with the mean value of 2.1) (Pajouhesh and Lenz, 2005).

## 4 Discussion

It is critical to find an effective treatment for inhibiting GSC stemness, which contributes to treatment failure and tumor progression (Osuka et al., 2021; Kim et al., 2024). Herein, DE-FeONPs was found to have the highest growth inhibitory potential on four GSCs (IC_50_ ≤ 70 μg/mL) and four corresponding GSCs-RR (IC_50_ ≤ 161 μg/mL), compared to TMZ (≤276 and ≤485 μg/mL, respectively) and other nanocomplexes. Furthermore, DE-FeONPs inhibited self-renewal and cancer repopulation capabilities of human and mouse GSCs and their RR (IC_50_ ≤ 116 μg/mL) more effectively than TMZ (IC_50_ ≤ 385 μg/mL). Up to 75 μg/mL of DE-FeONPs increased sensitivity of MGG18-RR and GSRR to TMZ by > 2 folds. The minimum concentration of DE-FeONPs which can increase the sensitivity of human MGG18-RR and mouse GSRR to TMZ and human MGG18-RR to radiotherapy (2Gy or 5 Gy) by ≥ 2 folds, was 75 μg/mL. Only the maximum dose of DE-FeONPs (300 μg/mL) could significantly improve the sensitivity of mouse GSRR to radiotherapy (2Gy or 5 Gy) by > 2 folds. Importantly, DE-FeONPs lowered the expression of all studied stemness genes and relative p-AKT protein expression level with higher efficacy than TMZ in both human and mouse GSCs and GSCs-RR.

The potent anti-stemness influence of DE-FeONPs is attributed to inhibiting ALDH1A1 and inducing lipid peroxidation-dependent ferroptosis (Abu-Serie, 2024). Since, the balance between reactive radical species and antioxidants is primarily responsible for therapeutic resistance in GBM (Zhuo et al., 2022), inducing ferroptosis could be one of the most promising treatments (Luo et al., 2022; Xue et al., 2024). Notably, GSCs have a higher capacity than normal brain cells for iron uptake by upregulating the expression of its receptor (Park et al., 2019). Interestingly, a previous study reported that TMZ can induce ferroptosis by upregulating the divalent metal transporter (DMT)1 expression for enhancing iron uptake and inhibiting nuclear factor erythroid related factor (Nrf)2-mediated GSH and GPX expression (Song et al., 2021). However, no discernible change in lipid peroxidation levels was observed in TMZ-treated wells versus the untreated wells (Figure 9B (I, II)). This is mainly attributed to the fact that these TMZ-treated spheres had no marked change in ALDH1A1 activity (Figure 8B). In line with this current result, previous studies demonstrated that expressions of ALDH1A1 as well as stemness genes (e.g., ABCG2 and NOTCH1) increased in response to TMZ treatment in GBM cells (Chua et al., 2008; Schäfer et al., 2012; Tango et al., 2018; Alves et al., 2021). Most importantly, Auffinger et al. (2014) declared critical data concerning that TMZ stimulates the conversion of non-GSCs to therapeutic resistant and invasive GSCs (*in vitro* and *in vivo*), explaining the high rate of cancer relapse after TMZ treatment (Auffinger et al., 2014). In line with previous studies, this study showed that ALDH1A1 activity not only sustains stemness redox homeostasis via detoxifying reactive aldehyde species (products of lipid peroxidation), but it also maintains the activity, expression, and stability of hall-markers of stemness phenotypes, including AKT, β-catenin (CTNNB1), ABCG2, and HIF-1A (Wang et al., 2020; Poturnajova et al., 2021). Hence, ALDH1A1 prevents TMZ- and radiation-induced DNA damage (i.e., resistance to chemotherapy and radiation by protecting DNA from genotoxicity) and mediates stemness and anti-apoptosis (Schäfer et al., 2012; Tango et al., 2018; Zhong et al., 2022). Therefore, patients with high ALDH1A1 expression level had a poorer prognosis and therapeutic response than those with low levels (Schäfer et al., 2012).

Diethyldithiocarbamate inhibits ALDH1A1 via its thiol affinity, forming a disulfide bond in the active site (Koppaka et al., 2012; Abu-Serie, 2018). In DE-FeO NPs-treated GSCs and GSC-RR, the inhibition of ALDH1A1 activity was associated with lowering ALDH1A1 gene expression. The latter could be attributed to suppressing the expression of β-catenin (CTNNB1), a key mediator for triggering ALDH1A1 transcription (Poturnajova et al., 2021). Previous study reported that disulfiram, the parent precursor of DE, inhibited MGMT activity in human glioblastoma cell lines (Paranjpe et al., 2014). This efficiency was potentiated when disulfiram was combined with copper, generating DE/Cu complex (Wang et al., 2022), with a more potent inactivation of proteasome, resulting in toxic protein accumulation, and subsequently improving sensitivity to TMZ and radiation (Hothi et al., 2012; Lun et al., 2016; Zhuo et al., 2022). The thiol affinity of DE also inactivates the antioxidant glutathione system (GPX4 and GSH), promoting ferroptosis that is triggered by accumulation of FeO NPs (Abu-Serie, 2023; Abu-Serie and Abdelfattah, 2023). Iron (Fe^+2^) not only initiates lipid peroxidation by reacting with hydrogen peroxides, forming hydroxyl radicals, but also mediates its autopropagation by converting lipid peroxides into peroxyl and alkoxyl radicals (Luo et al., 2022).

As declared in this study, DE-FeO NPs caused severe suppression in ALDH1A1 activity, GSH level, and GPX4 activity (Figures 8B–D), resulting in a significant increase in ROS and lipid peroxidation (Figures 9A, B). These results indicate the incidence of efficient ferroptosis-mediated chemosensitivity and radiosensitivity (Figures 6B, C) and stemness halting, which was confirmed by suppressing stemness genes and p-AKT level. ALDH1A1 as well as the studied genes (including, PROM1, NOTCH1, CTNNB1, nestin, and EGFR), which mediate stemness and therapeutic resistance, activate the PI3K/p-AKT pathway (Vassalli, 2019; Eckerdt et al., 2020; Tang et al., 2021; Tsai et al., 2022). p-AKT enhances the expression of the main stemness-regulating transcription factors (NANOG, OCT-4, and SOX2), anti-apoptotic proteins, and HIF1A-mediated hypoxia, promoting self-renewal, tumor progression, chemoresistance and radioresistance (Li et al., 2016; Shin et al., 2019; Singh et al., 2021). HIF1A not only mediates radioresistance but also stimulates, together with β-catenin and ZEB1, TMZ resistance via inducing MGMT overexpression (Alves et al., 2021; Chien et al., 2021; Singh et al., 2021; Tang et al., 2021). Additionally, the Sonic Hedgehog pathway enhances the overexpression of MGMT and drug efflux transporters (ABCB1, ABCC1, and ABCG2) (Tang et al., 2021). Accordingly, downregulation of these above-mentioned genes resulted in a drastic collapse in stemness features (self-renewal, cancer repopulation, and therapeutic resistance) of DE-FeONPs-treated GSCs and GSCs-RR, compared to TMZ-treated spheres.

Besides the therapeutic resistance, the blood brain barrier is viewed as another limitation for the remedy’s effect on GBM by preventing the effective dose from reaching GSCs. Interestingly, the DE-FeONPs exhibited the ideal partitioning for crossing the blood brain barrier as declared by experimental determination of LogP.

## 5 Conclusion

The nanocomplex of DE-FeONPs exhibited the strongest growth inhibitory potential on human and mouse GSCs and GSCs-RR, compared to TMZ, DE, metal oxide NPs, and other nanocomplexes (DE-Cu_4_O_3_ NPs and DE-Cu_2_O NPs). Moreover, DE-FeONPs inhibited self-renewal, cancer repopulation, and chemoresistance and radioresistance of human MGG18 and mouse GS as well as their corresponding GSCs-RR (MGG18-RR and GSRR) more effectively than TMZ. These were associated with a higher repression of stemness gene expression levels and relative p-AKT/AKT ratio in DE-FeONPs-treated spheres than TMZ-treated spheres. This potent anti-stemness effect of DE-FeONPs is mainly attributed to DE’s effect on lowering anti-ferroptotic factors (ALDH1A1 activity, GSH level, and GPX4 activity), which enhanced the inducible ferroptosis impact triggered by FeONPs. Ferroptosis was more powerful in DE-FeONPs-treated spheres than in TMZ-treated spheres, as evidenced by extreme elevations in cellular ROS and lipid peroxidation contents. Importantly, experimental Log*P* studies predicted the blood brain permeability of this nanocomplex. Accordingly, DE-FeONPs could represent a novel promising effective remedy for GBM by targeting therapeutic resistance GSCs. Animal models of GBM will be needed in the future to study its potential and pharmacokinetics in depth.

## Data Availability

The original contributions presented in the study are included in the article/Supplementary Material, further inquiries can be directed to the corresponding author.

## Abbreviations

ABC, ATP-binding cassette; ALDH1A1, Aldehyde dehydrogenase 1A1; CSCs, Cancer stem cells; DE, Diethyldithiocarbamate; EGFR, Epidermal growth factor receptor; GBM, Glioblastoma; GPX4, Glutathione peroxidase; GSCs, Glioma stem cells; GSH, Glutathione; GSCs, Glioblastoma stem cells; GSCs-RR, Glioblastoma stem cells-radioresistance; HIF, Hypoxia-inducible factor; Log*P*, Oil/water partition coefficient; MGMT, O^6^-methylguanine-DNA methyltransferase; NPs, Nanoparticles; PDGF-GSC, Platelet derived growth factor-induced glioma stem cells; ROS, Reactive oxygen species; TMZ, Temozolomide.
