# Supplementary material for: Diethyldithiocarbamate-ferrous oxide nanoparticles inhibit human and mouse glioblastoma stemness: aldehyde dehydrogenase 1A1 suppression and ferroptosis induction
Source: Front Pharmacol. 2024 Apr 24;15:1363511. doi: 10.3389/fphar.2024.1363511 (PMC11076782; doi:10.3389/fphar.2024.1363511)
Supplement: Supplementary file 1 [file Table1.docx]

Supplementary Material

| Gene name | Sequences | Accession number | R^2^ | Efficiency |
| --- | --- | --- | --- | --- |
| ALDH1A1 | F: TGGACCAGTGCAGCAAATCA  R: ACGCCATAGCAATTCACCCA | NM_000689 | 0.980 | 1.933 |
| ABCB1 | F: AGATAAAAGAGAGGTGCAACGG  R: CCTGTGGCAAAGAGAGCGAA | NM_000927 | 0.988 | 1.925 |
| ABCC1 | F: GAGCTGGAACCTGACAGCAT  R: TCGGGGATGGAGAAGGTGAT | NM_004996 | 0.981 | 2.041 |
| ABCG2 | F: CTGTTTTGTGTTTATGATGGTCTGT  R: TGCTGCAAAGCCGTAAATCC | NM_004827 | 0.984 | 1.929 |
| CD44 | F: GGGATATCGCCAAACACCCA  R: TGGATGGCTGGTATGAGCTG | NM_000610 | 0.988 | 1.992 |
| PROM1 | F: GAGCTAAGGGAAGGGCGG  R: TTCTGTCTGAGGCTGGCTTG | NM_006017 | 0.984 | 1.912 |
| NOTCH1 | F: CAGACTATGCCTGCAGCTGTG  R: CTGGCACGATTTCCCTGACC | NM_017617 | 0.996 | 2.046 |
| CTNNB1 | F: GGCTACTCAAGCTGATTTGATGG  R: AAGACTGTTGCTGCCAGTGA | NM_001098209 | 0.991 | 1.902 |
| NANOG | F: AATGGTGTGACGCAGGGATG  R: TGCACCAGGTCTGAGTGTTC | NM_024865 | 0.996 | 2.081 |
| OCT-4 | F: AGCACTTCTGTCATGCTGGA  R: AGCACCTTCTATAAGCCAGCG | NM_002701 | 0.995 | 1.945 |
| SOX2 | F: CATGAAGGAGCACCCGGATT  R: TAACTGTCCATGCGCTGGTT | NM_003106 | 0.982 | 1.906 |
| Nestin | F: CCACCCTGCAAAGGGAATCT  R: GGTGAGCTTGGGCACAAAAG | NM_006617 | 0.999 | 1.972 |
| EGFR | F: GTGGGATAGCCCTCCCTGTA  R: GCACCTTTTGCAGGGCATTT | NM_005228 | 0.998 | 2.030 |
| MGMT | F: ACGCTGCCCTTGCTCTATTT  R: AGCTTTCTAGTGTGGACGGC | NM_002412 | 0.980 | 1.970 |
| HIF1A | F: AGAGGTTGAGGGACGGAGAT  R: TCCGACATTGGGAGCTCATT | NM_001530 | 0.995 | 1.941 |
| ZEB1 | F: GCTGTTTCAAGATGTTTCCTTCCA  R: GCCTATGCTCCACTCCTTGC | NM_030751 | 0.992 | 1.945 |
| β-actin | F: GTCATTCCAAATATGAGATGCGT  R: GCTATCACCTCCCCTGTGTG | NM_001101 | 0.995 | 1.940 |

**Supplementary Table 1**. Primers (forward “F” and reverse “R”, accession number, Pearson’s coefficient (R^2^), and amplification efficiency) of the used human genes
