# Supplementary material for: Diethyldithiocarbamate-ferrous oxide nanoparticles inhibit human and mouse glioblastoma stemness: aldehyde dehydrogenase 1A1 suppression and ferroptosis induction
Source: Front Pharmacol. 2024 Apr 24;15:1363511. doi: 10.3389/fphar.2024.1363511 (PMC11076782; doi:10.3389/fphar.2024.1363511)
Supplement: Supplementary file 2 [file Presentation1.PPTX]

## Slide 1
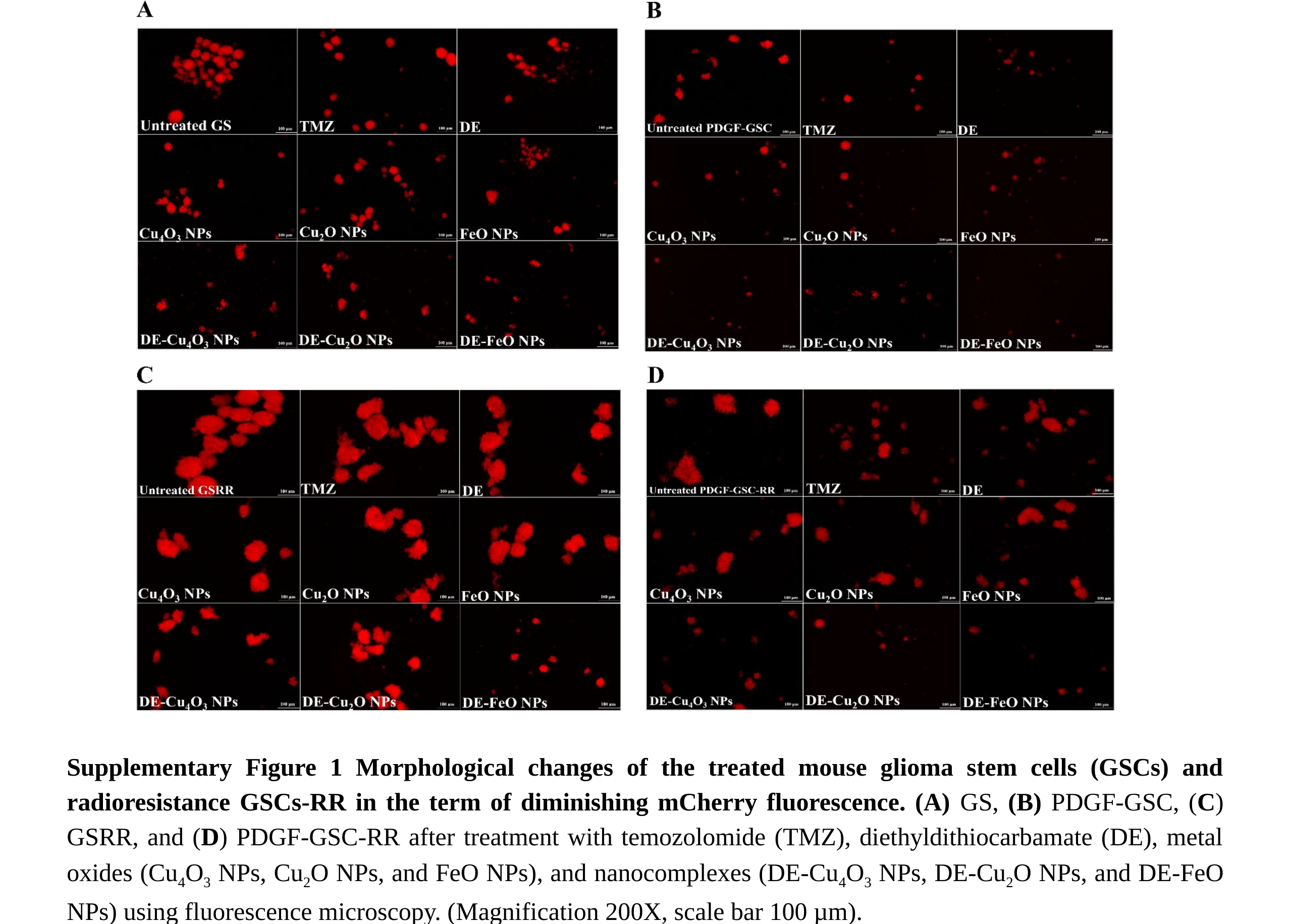

Supplementary Figure 1 Morphological changes of the treated mouse glioma stem cells (GSCs) and radioresistance GSCs-RR in the term of diminishing mCherry fluorescence. (A) GS, (B) PDGF-GSC, (C) GSRR, and (D) PDGF-GSC-RR after treatment with temozolomide (TMZ), diethyldithiocarbamate (DE), metal oxides (Cu4O3 NPs, Cu2O NPs, and FeO NPs), and nanocomplexes (DE-Cu4O3 NPs, DE-Cu2O NPs, and DE-FeO NPs) using fluorescence microscopy. (Magnification 200X, scale bar 100 µm).
