# Supplementary material for: Diethyldithiocarbamate-ferrous oxide nanoparticles inhibit human and mouse glioblastoma stemness: aldehyde dehydrogenase 1A1 suppression and ferroptosis induction
Source: Front Pharmacol. 2024 Apr 24;15:1363511. doi: 10.3389/fphar.2024.1363511 (PMC11076782; doi:10.3389/fphar.2024.1363511)
Supplement: Supplementary file 3 [file Table2.docx]

Supplementary Material

**Supplementary Table 2**. Primers (forward “F” and reverse “R”, accession number, Pearson’s coefficient (R^2^), and amplification efficiency) of the used mouse genes

| Gene name | Sequences | Accession number | R^2^ | Efficiency |
| --- | --- | --- | --- | --- |
| ALDH1A1 | F: TAAAGCTGGGAAATGCCCCC  R: TGCAAACCTCTCCCTTTGCT | NM_013467 | 0.995 | 1.919 |
| ABCB1 | F: CAGTGGCTCTTGAAGCCGTA  R: TCCTTCGCAAAGTCAGCCAA | NM_011075 | 0.988 | 2.028 |
| ABCC1 | F: TGACTCTCAAGGGCTCCGT  R: GGGCACAGGCTTCCATAACT | NM_008576 | 0.983 | 1.903 |
| ABCG2 | F: TGAATAGCTGACCATCAGTGCC  R: GCTGCTTAATCTGGCCTCACA | NM_011920 | 0.988 | 2.012 |
| CD44 | F: GATGCCTGTTGCAAGTACTCC  R: AGCACTGGTGCTGATTCTGT | NM_009851 | 0.990 | 1.903 |
| PROM1 | F: AATTAAGTGGAAGGAGCCCAG  R: TCTCCAAGGTGGTCATTCACT | NM_008935 | 0.993 | 2.017 |
| NOTCH1 | F: CACCAGGGTGGTCAGGAAAA  R: GGGCAGCGACAGATGTATGA | NM_008714 | 0.998 | 1.921 |
| CTNNB1 | F: AGGTCAGCTTGAGTAGCCATTG  R: CGTCAGCTCGTGTCCTGTGA | NM_007614 | 0.991 | 1.931 |
| NANOG | F: GGCTCACTTCCTTCTGACTTCT  R: CTCATGTCAGTGTGATGGCG | NM_028016 | 0.994 | 1.928 |
| OCT-4 | F: GGGCTAGAGAAGGATGTGGTTC  R: GAAAGGTGTCCCTGTAGCCTC | NM_013633 | 0.997 | 2.032 |
| SOX2 | F: GGAGGAGAGCGCCTGTTTTT  R: CTGGCGGAGAATAGTTGGGG | NM_011443 | 0.982 | 1.944 |
| Nestin | F: AAGGGCCACTCCCTTCTCTA  R: AGATTCTTCCCCGACGCAAC | NM_016701 | 0.991 | 1.930 |
| EGFR | F: GCCAGGTCTTCAAGGATGTGA  R: GGTGAGAGGGGAGTCAGAGAT | NM_007912 | 0.996 | 1.919 |
| MGMT | F: GCAGTGAGGCTGTGTTTGAGA  R: TTTGCAGGTCTCAGCCATTCT | NM_008598 | 0.980 | 1.989 |
| HIF1A | F: AGGATGAGTTCTGAACGTCGAAA  R: GGGGAAGTGGCAACTGATGA | NM_010431 | 0.997 | 2.036 |
| ZEB1 | F: TCCCCAGCTCACAATAAACG  R: GCTCTAGGATGTAATGCCCAC | NM_011546 | 0.989 | 1.934 |
| β-actin | F: CGCCACCAGTTCGCCA  R: CTTTGCACATGCCGGAGCC | NM_007393 | 0.995 | 1.992 |
